# PFKFB3 blockade inhibits hepatocellular carcinoma growth by impairing DNA repair through AKT

**DOI:** 10.1038/s41419-018-0435-y

**Published:** 2018-03-20

**Authors:** Wen-Kai Shi, Xiao-Dong Zhu, Cheng-Hao Wang, Yuan-Yuan Zhang, Hao Cai, Xiao-Long Li, Man-Qing Cao, Shi-Zhe Zhang, Kang-Shuai Li, Hui-Chuan Sun

**Affiliations:** 1 Liver Cancer Institute, Zhongshan Hospital, Fudan University, 200032 Shanghai, China; 2 Key Laboratory of Carcinogenesis and Cancer Invasion (Fudan University), Ministry of Education, 200032 Shanghai, China; 3 Department of Liver Surgery, Fudan University Shanghai Cancer Center, Cancer Hospital, 200032 Shanghai, China

## Abstract

Overexpression of 6-phosphofructo-2-kinase/fructose-2,6-biphosphatase 3 (PFKFB3), a key molecule of glucose metabolism in cytoplasm, has been found in various tumors. Emerging evidence has suggested that PFKFB3 is also located in the nucleus and apparent in regulatory functions other than glycolysis. In this study, we found that PFKFB3 expression is associated with hepatocellular carcinoma (HCC) growth and located mainly in the nucleus of tumor cells. PFKFB3 overexpression was associated with large tumor size (*p* = 0.04) and poor survival of patients with HCC (*p* = 0.027). Knockdown of PFKFB3 inhibited HCC growth, not only by reducing glucose consumption but also by damaging the DNA repair function, leading to G2/M phase arrest and apoptosis. In animal studies, overexpression of PFKFB3 is associated with increased tumor growth. Mechanistically, PFKFB3 silencing decreased AKT phosphorylation and reduced the expression of ERCC1, which is an important DNA repair protein. Moreover, PFK15, a selective PFKFB3 inhibitor, significantly inhibited tumor growth in a xenograft model of human HCC. PFKFB3 is a potential novel target in the treatment of HCC.

## Introduction

Primary liver cancer is the second most common cause of cancer-related death in the world, and 90% of liver cancers are hepatocellular carcinoma (HCC)^1^. Although curative treatment provides long-term survival for patients during early stage HCC (Barcelona clinic liver cancer (BCLC) stage 0 and A), approximately 70% of patients have the advanced stage; therefore, they are not amenable for curative treatment, and the survival of these patients is poor^2^. Survival associated with systemic treatment, such as sorafenib, for advanced stage cancer patients is approximately 3 months^3^. Hence, it is important to find a novel treatment for HCC.

Accumulating evidence suggests that the continuous activation of aerobic glycolysis (Warburg effect) plays a vital role in tumor development^4^ and the many altered gene expressions accompanied by aerobic glycolysis in tumor development^5^. One of the altered genes is 6-phosphofructo-2-kinase/fructose-2,6-bisphosphatase 3 (PFKFB3), which significantly accelerates the glycolysis rate and is expressed in rapidly multiplying cells and various human cancers^6,7^. In a previous report, PFKFB3 was overexpressed in cancer cells and associated with cancer progression^8^. In breast and ovarian cancer cell line models, the PFKFB3 inhibitor 3-PO suppresses glycolytic flux and tumor growth^9^. In a study of head and neck squamous cell carcinoma, blocking PFKFB3 suppressed tumor growth and metastasis mediated by suppressing glycolysis^10^.

However, a report revealed that in some cancer cells (Hela, HCT116, and MDA-MB-231), PFKFB3 localized not only in the cytoplasm (the site of glycolysis) but also in the nucleus, and overexpressed PFKFB3 increased during cell proliferation without changes in glucose metabolism^11^. Furthermore, PFKFB3 promoted cell cycle progression and suppressed apoptosis via Cdk1-mediated phosphorylation of p27^12^, and MAPK increased the PFKFB3 transcript to accelerate cell proliferation^13^. Nevertheless, the specific location and function of PFKFB3 in HCC cells are not known.

We report that PFKFB3 was mainly located in the nucleus in tumor cells and that PFKFB3 overexpression was associated with tumor progression by directly regulating cell proliferation. Moreover, PFK15, a selective PFKFB3 inhibitor, significantly inhibited tumor growth.

## Results

### Expression of PFKFB3 in HCC cells and correlated with the survival of HCC patients

**Fig. 1:**
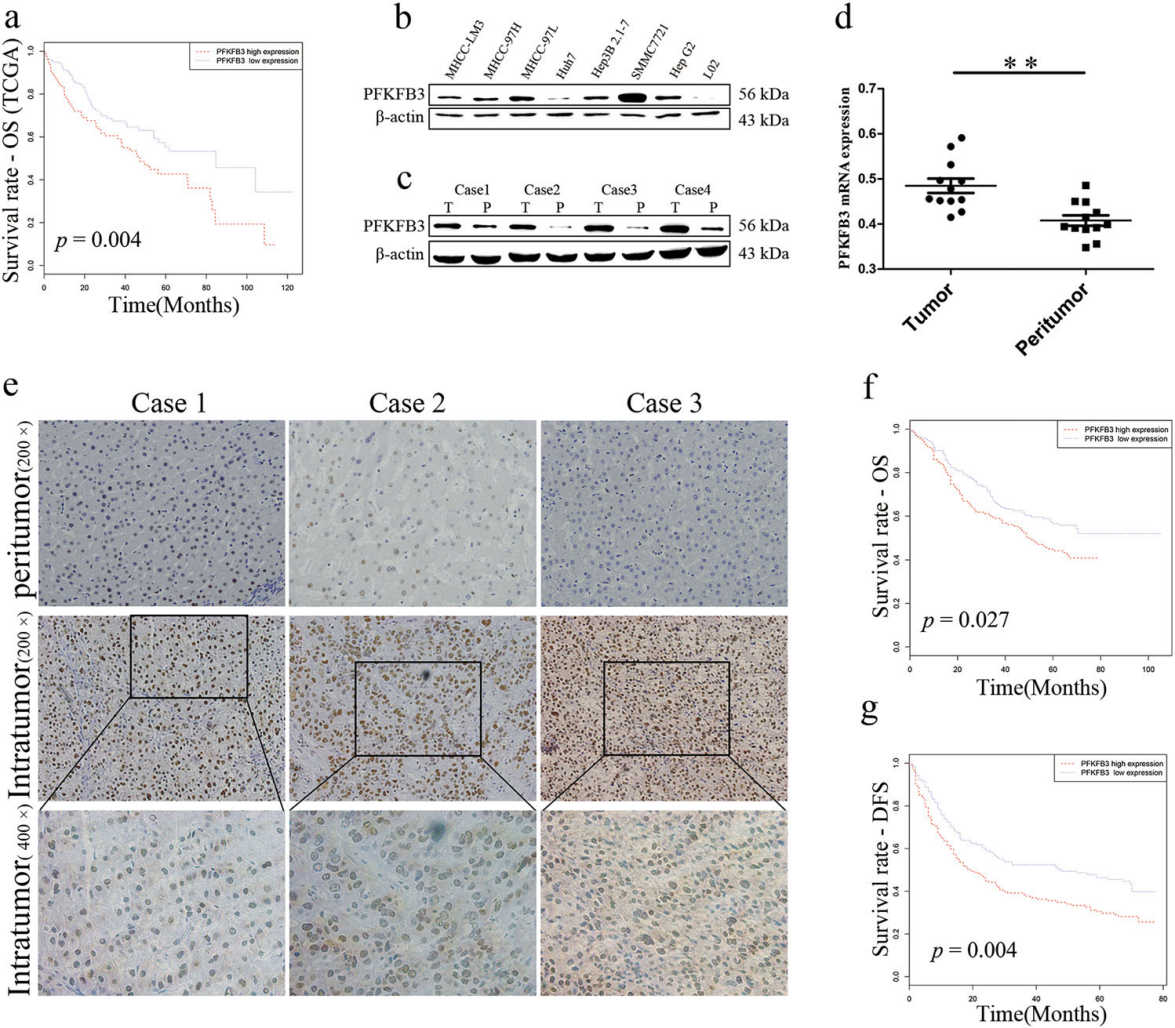
PFKFB3 overexpressed in liver cancer cells and correlated with poor prognosis in HCC patients. **a** Kaplan–Meier analysis for overall survival (OS) of patients based on the data of TCGA. The OS of the PFKFB3 high expression group was worse than that of the PFKFB3 low expression group (*p* = 0.004). **b** Western blot analysis of PFKFB3 protein expression in liver cancer cell lines and normal hepatocyte cell line (L02). The PFKFB3 expressed higher in liver cancer cell lines. **c** Western blot analysis of PFKFB3 protein expression in paired human hepatocellular carcinoma (HCC) and peritumor tissue (T = Tumor tissue, P = Peritumor tissue). The PFKFB3 protein expressed higher in tumor tissues. **d** qRT-PCR expression analysis of PFKFB3 mRNA in paired human HCC and peritumor tissue (*n* = 12). The PFKFB3 mRNA expressed higher in tumor tissues. **e** Immunohistochemistry (IHC) staining of paired human HCC and peritumor tissue. PFKFB3 protein expressed higher in tumor tissues and located mainly in nucleus in human liver cancer tissue. **f** Kaplan–Meier analysis for OS based on the data of our center. The OS (*p* = 0.027) of the high expression group were worse than that of the PFKFB3 low expression group. **g** Kaplan–Meier analysis for disease-free survival (DFS) based on the data of our center. The DFS (*p* = 0.004) of the high expression group were worse than that of the PFKFB3 low expression group. **p < 0.01

To explore the clinical significance of PFKFB3 in HCC, we first performed a Kaplan–Meier survival analysis based on the data of The Cancer Genome Atlas (TCGA), which included 374 HCC patients divided into two groups with high or low expressions of PFKFB3 according to the median value of PFKFB3 expression. Overall survial (OS) for patients with high PFKFB3 expression was poorer than that for patients with low PFKFB3 expression (5-year survival rate: 39.4% vs. 53.3%; *p* = 0.004) (Fig. 1a).

Then, we examined PFKFB3 expression in human HCC tissues and cell lines. PFKFB3 expression was detected in seven HCC cell lines and a normal hepatocyte cell line (L02) using Western blot. The results showed that PFKFB3 expression was higher in seven HCC cell lines compared with that in L02 assayed by Western blot. Among seven HCC cell lines, the highest expression of PFKFB3 was observed in SMMC-7721, and the lowest expression of PFKFB3 was observed in Huh7 (Fig. 1b). Therefore, PFKFB3 expression in SMMC-7721 was knocked down and was overexpressed in Huh7 in the following experiments.

PFKFB3 expression was further analyzed by quantitative polymerase chain reaction (qPCR) and Western blot in paired tumor (T) and peritumor (P) liver tissues from 12 patients with HCC. The results showed that PFKFB3 expression was higher in tumor tissues compared with paired peritumor liver tissues (Fig. 1c, d).

To further explore the clinical significance of PFKFB3 in HCC, we performed immunohistochemistry staining (Fig. 1e) on a tissue microarray that contained HCC and peritumor liver tissues from 287 HCC patients. The results showed that higher expression of PFKFB3 was associated with larger tumor size (*p = *0.04) (Table 1), poorer OS (*p* = 0.027) (Fig. 1f), and disease-free survival (DFS) (*p* = 0.004) (Fig. 1g). Multi-variant analysis showed that higher expression of PFKFB3 was an independent prognostic factor for OS of HCC patients (hazard ratio (HR), 1.430; 95% confidence interval (CI), 1.204 to 1.997; *p* = 0.026) and DFS (HR, 1.426; 95% CI, 1.057 to 1.923; *p* = 0.05) (Table 2). It was noted that PFKFB3 mainly located in the nucleus of tumor cells in human HCC specimens (Fig. 1e). Aerobic glycolysis undergoes cytoplasm, so we hypothesized that PFKFB3 mainly regulated HCC growth in the nucleus of tumor cells.

**Table 1:** Relationship between intratumor PFKFB3 and clinicopathologic features

Variable		Intratumor PFKFB3		
		Low expression	High expression	*p*
Age (years)		53.50	50.95	0.438
Gender	Female	21	27	0.356
	Male	122	117	
HBsAg	Negative	23	18	0.386
	Positive	120	126	
Cirrhosis	No	113	106	0.281
	Yes	30	38	
AFP	≤20 ng/ml	49	34	0.047
	>20 ng/ml	94	110	
Size (cm)		5.36	5.67	0.040
Number	Single	125	123	0.622
	Multiple	18	21	
Thrombus	No	93	78	0.061
	Yes	50	66	
	I	85	68	0.046
TNM	II	38	58	
	III	20	18	
Encapsulation	Yes	72	70	0.768
	No	71	74	
Grade	I-II	105	97	0.260
	III-IV	38	47

**Table 2:** Univariate and multivariate analyses of factors associated with survival and recurrence

Clinicopathological Factors		Overall survival				Disease-free survival		
	Univariate *P*	Multivariate			Univariate *P*	Multivariate		
		Hazard ratio	95% CI	*P*		Hazard ratio	95% CI	*P*
Age: ≤50 vs. >50	0.915			NA	0.254			NA
Gender: female vs. male	0.387			NA	0.198			NA
Cirrhosis: no vs. yes	0.429			NA	0.429			NA
HBsAg: negative vs. positive	0.395			NA	0.501			NA
AFP: ≤20 vs. >20 ng/ml	0.105			NA	0.065			NA
Size: ≤ 5 vs. >5 cm	0.000	2.165	1.497 to 3.130	0.000	0.000	1.737	1.253–2.410	0.001
Number: single vs. multiple	0.009			NS	0.000			NS
Thrombus: no vs. yes	0.000			NS	0.000			NS
TNM: I vs. II vs. III	0.000	1.817	1.118 to 2.952	0.016	0.000	1.623	1.052–2.502	0.028
Encapsulation: yes vs. no	0.235			NS	0.085			NS
Grade: I-II vs. III-IV	0.012	1.432	1.001 to 2.049	0.049	0.004	1.513	1.098–2.085	0.011
Intratumor PFKFB3	0.026	1.430	1.204 to 1.997	0.036	0.005	1.426	1.057–1.923	0.020

*NA* not applicable, *NS* not significant

### PFKFB3 expression promoted HCC cell proliferation

**Fig. 2:**
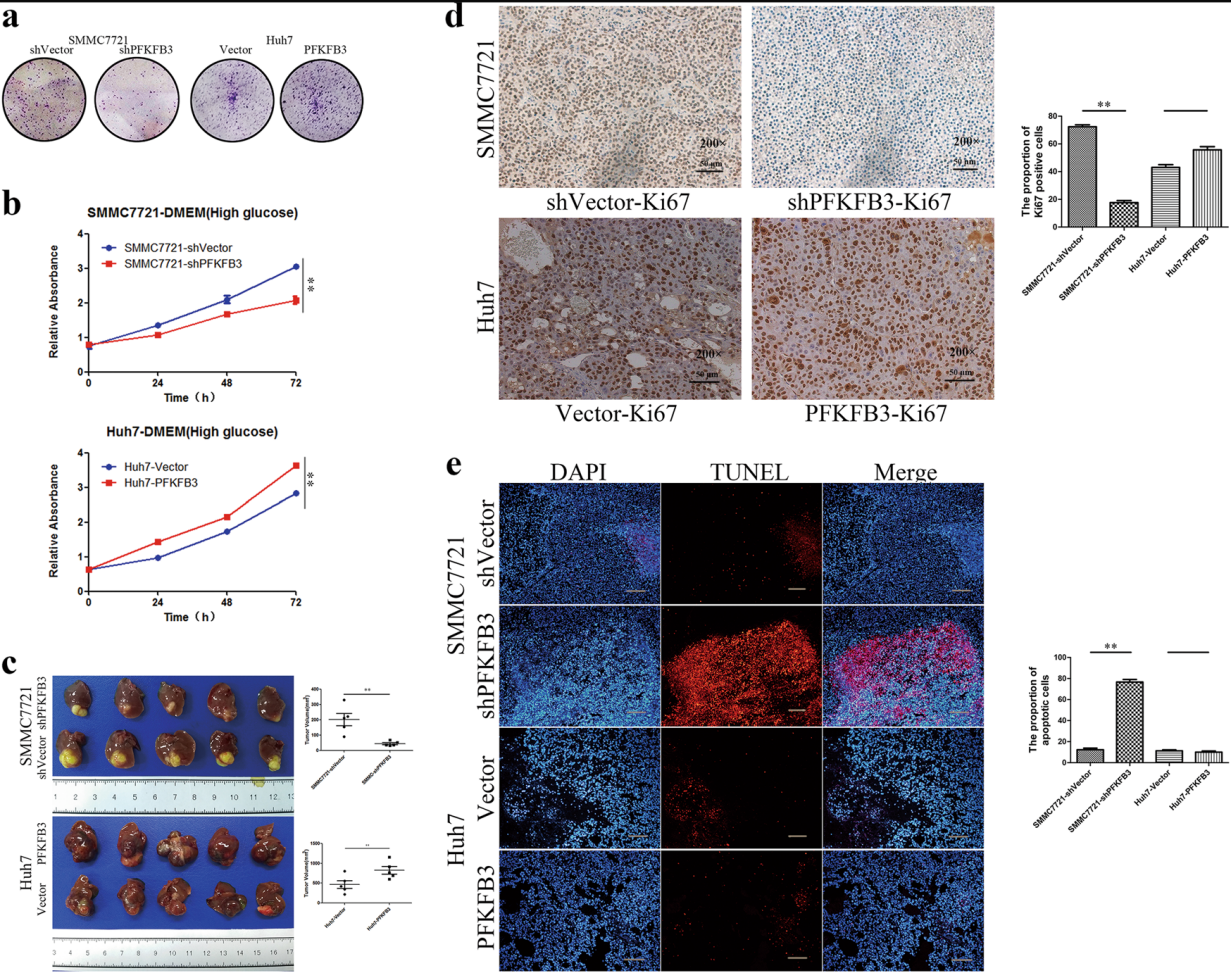
PFKFB3 expression in HCC cells promoted tumor growth in vitro and in vivo. **a** Clone formation of SMMC7721-shPFKFB3 cells and Huh7-PFKFB3 cells compared with their vector control. PFKFB3 promoted the cells clone formation in vitro. **b** CCK8 assay for cell proliferation of SMMC7721-shPFKFB3 cells and Huh7-PFKFB3 cells compared with their vector control. PFKFB3 promoted the liver cancer cells proliferation in vitro. **c** Comparison of tumor sizes for the SMMC7721-shPFKFB3 cells group and SMMC7721-shVector cells group (45.3 ± 14.9 mm^3^ vs. 201.9 ± 88.6 mm^3^; *p* = 0.02), and comparison of tumor sizes for the Huh7-PFKFB3 cells group and Huh7-Vector cells group (825.6 ± 217.9 mm^3^ vs. 467.8 ± 221.9 mm^3^; *p* = 0.033). **d** Representative immunohistochemistry of liver cancer for the expression of Ki67 from Balb/c nu/nu mice orthotopically implanted with SMMC7721 or Huh7 cells. Ki67 expressed higher in PFKFB3 high expression tumor. (Magnification ×200). **e** Representative TUNEL fluorescence of liver cancer from Balb/c nu/nu mice orthotopically implanted with SMMC7721 or Huh7 cells. The apoptosis rate of cells was higher in PFKFB3 low expression tumor. (Magnification ×200). *p < 0.05, **p < 0.01

We studied whether the expression of PFKFB3 affected HCC cell proliferation. First, we constructed a stable PFKFB3 overexpressed cell line (Huh7-PFKFB3) and PFKFB3 knockdown cell line (SMMC7721-shPFKFB3), which were confirmed with Western blot and qPCR assays (supplementary figure 1A, B). Clone formation assays and CCK8 proliferation assays showed that clone formation and cell proliferation were decreased in SMMC7721-shPFKFB3 cells compared with SMMC7721-shVector (Fig. 2a, b). In contrast, Huh7-PFKFB3 cells, compared with Huh-Vector, significantly increased the colony-forming ability and cell proliferation (Fig. 2a, b).

To further explore the effects of PFKFB3 expression on HCC growth, SMMC7721, and Huh7 cells transfected with lentiviral vectors were orthotopically implanted into Balb/c nu/nu mice (*n* = 5 for each group). The tumor sizes of the SMMC7721-shPFKFB3 group were smaller than those of the SMMC7721-vector group (45.3 ± 14.9 mm^3^ vs. 201.9 ± 88.6 mm^3^; *p* = 0.02) (Fig. 2c), and the tumor sizes of the Huh7-PFKFB3 group were larger than those of Huh7-vector group (825.6 ± 217.9 mm^3^ vs. 467.8 ± 221.9 mm^3^; *p* = 0.033) (Fig. 2c). Moreover, we analyzed Ki67 expression as a proliferation marker and found that proliferation decreased in the SMMC7721-shPFKFB3 tumors compared with that in the SMMC7721-shVector tumors, whereas the proliferation of the Huh7-PFKFB3 tumors was increased compared with that of the Huh7-vector tumors (Fig. 2d). Furthermore, we conducted a TUNEL assay to detect apoptosis in tumor specimens from the xenograft models and found that apoptosis was decreased in the Huh7-PFKFB3 tumors compared with that in the Huh7-vector tumors (Fig. 2e); however, it was increased in the SMMC7721-shPFKFB3 tumors compared with that in the SMMC7721-shVector tumors (Fig. 2e).

### PFKFB3 inhibition led to G2/M phase arrest and apoptosis of HCC cells

**Fig. 3:**
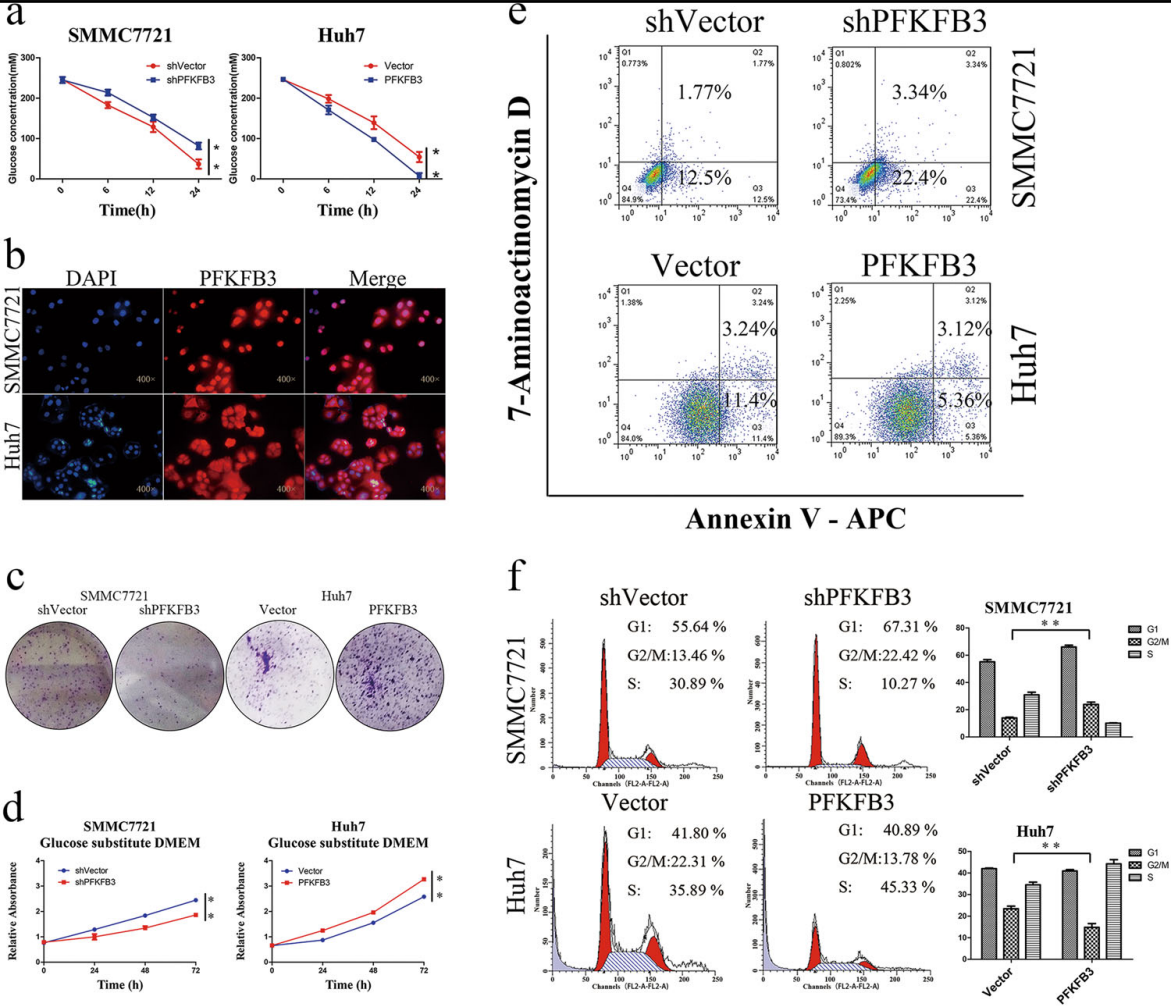
PFKFB3 inhibition led to G2/M phase arrest and apoptosis of HCC cells. **a** Glucose consumption of different PFKFB3 expressions of SMMC7721 and Huh7 cells. High expression cells consumoted more glucose. **b** Immunofluorescence stain of PFKFB3 in SMMC7721 and Huh7 cells. PFKFB3 located both in nucleus and cytoplasm. **c** Clone formation of SMMC7721-shPFKFB3 cells and Huh7-PFKFB3 cells compared with their vector control in glucose substitute medium. PFKFB3 promoted the cells clone formation eliminating the effect of glucose in vitro. **d** CCK8 assay for cell proliferation of SMMC7721-shPFKFB3 cells and Huh7-PFKFB3 cells compared with their vector control in glucose substitute medium. PFKFB3 promoted the liver cancer cells proliferation eliminating the effect of glucose in vitro. **e** Flow cell apoptosis detection for cell apoptosis rates of SMMC7721-shPFKFB3 cells and Huh7-PFKFB3 cells compared with their vector control in glucose substitute medium. PFKFB3 decreased the apoptosis rate of liver cancer cells eliminating the effect of glucose in vitro. **f** Flow cytometry cycle detection of the cell cycle ratio of SMMC7721-shPFKFB3 cells and Huh7-PFKFB3 cells compared with their vector control in glucose substitute medium. PFKFB3 knockdown increased the proportion of G2/M phase. **p < 0.01

To explore the role of PFKFB3 in glucose metabolism, we examined glucose consumption in Huh7 and SMMC7721 cell lines. The results showed that glucose consumption in Huh7-PFKFB3 cells was higher than that in Huh7-vector cells (Fig. 3a), and it was lower in the SMMC7721-shPFKFB3 cells than that in the SMMC7721 cells (Fig. 3a).

We found that PFKFB3 can be detected in the nucleus of HCC cells in the surgical specimens (Fig. 1e) and Huh7 and SMMC7721 cells (Fig. 3b). And to eliminate the effect of antibody specificity, we conducted the immunofluorescence to detect the change when knockdown of PFKFB3 in SMMC7721. And we found that knockdown of PFKFB3 reduced the nuclear staining, which eliminated the effect of antibody specificity (supplementary figure2). To study whether PFKFB3 directly promotes cell proliferation, we tried to abrogated the effects of PFKFB3 on glucose metabolism. During glucose metabolism, glucose is to be converted to pyruvate, which enters the tricarboxylic acid cycle or converts to lactic acid and produces ATP^14^. PFKFB3 is a key enzyme in the conversion of glucose to pyruvate, and sodium pyruvate is an important alternative carbon source in cell culture^15^. We used sodium pyruvate as an alternative carbon source to bypass the function of PFKFB3 on glucose metabolism. As expected, cell apoptosis rate increased and cell proliferation was significantly suppressed when cultured in the glucose-free medium (supplementary figure 3A, B). When cells were cultured in the glucose-free medium supplemented with sodium pyruvate (100 mM, glucose substitute medium), cell proliferation, apoptosis, and cell cycles were not different from those in the glucose-containing medium (supplementary figure 3A, B, C). Then, we used the glucose substitute medium to conduct an experiment to eliminate the influence of glucose metabolism.

We found that clone formation and cell proliferation were decreased in SMMC7721-shPFKFB3 cells compared with SMMC7721-shVector cells when the cells were cultured in the glucose substitute medium. In contrast, Huh7-PFKFB3 cells presented increased colony-forming and cell proliferation abilities compared with the control group (Fig. 3c, d). This suggested that, in addition to its role in glucose metabolism, PFKFB3 directly regulated cell growth.

Flow cytometry assays showed that the cell apoptosis rate and G2/M phase ratio were increased in SMMC7721-shPFKFB3 cells compared with vector control cells when they were cultured in the glucose substitute medium. In contrast, Huh7-PFKFB3 cells presented decreased apoptosis rates and G2/M phase ratios compared with vector control cells (Fig. 3e, f).

### PFKFB3 knockdown inhibited DNA repair via the AKT/ERCC1 pathway

**Fig. 4:**
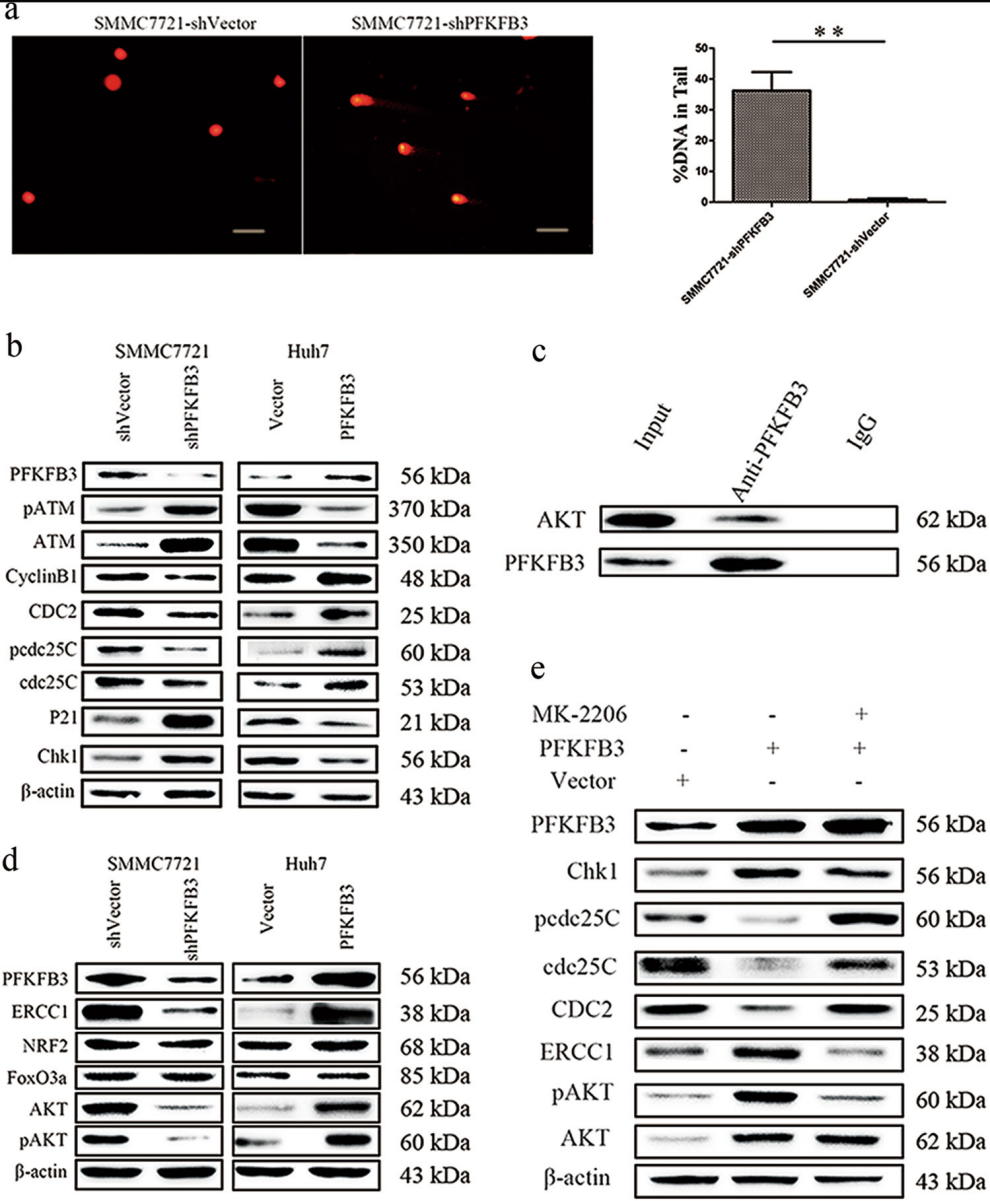
PFKFB3 knockdown inhibited DNA repair via the AKT/ERCC1 pathway. **a** Comet assay for DNA damage of SMMC7721-shPFKFB3 and SMMC7721-shVector. In SMMC7721-shPFKFB3 cells, DNA damage presented more compared with SMMC7721-shVector cells. **b** The ATM/Chk1/cdc25C signaling pathway-associated proteins were detected by Western blot in SMMC7721-shPFKFB3 and Huh7-PFKFB3 compared with their vector control. **c** Immunoprecipitation assay was conducted for SMMC7721 cells, and IgG was used as a control. AKT was immunoprecipitated by anti-PFKFB3 antibody. **d** The PFKFB3, AKT, pAKT, and ERCC1 were detected by Western blot in SMMC7721-shPFKFB3 and Huh7-PFKFB3 compared with their vector control. **e** Inhibited AKT phosphorylated by MK-2206 in Huh7-PFKFB3 cells. PFKFB3 expression was not increased but ERCC1 expression was decreased in Huh7-PFKFB3 cells, and the ATM/Chk1/cdc25C signaling pathway had no significant change. **p < 0.01

The main reason leading to G2/M phase arrest is activation of cell cycle checkpoint genes, which comprise a number of PI3 kinase genes and are usually trigged by DNA damage^16^. Therefore, we performed a comet assay and found that DNA fragments were more often found in SMMC7721-shPFKFB3 cells than in SMMC7721-shVector, suggesting that PFKFB3 knockdown led to increased DNA damage (Fig. 4a).

To elucidate the molecular mechanisms of PFKFB3 during G2/M phase arrest, an Agilent cDNA microarray analysis was conducted for SMMC7721-shPFKFB3 and SMMC7721-shVector cells (GSE106603). The results demonstrated 2178 differentially expressed genes (DEGs), including 1473 upregulated genes and 705 downregulated genes (|log FC| ≥ 2) that were identified. A KEGG analysis showed that PI3K-Akt signaling pathway is the most enriched signaling pathway (*p* < 0.001) (supplementary figure 4). GO analysis showed that DNA repair is one of the biological processes most affected by PFKFB3 knockdown in SMMC-7721 cells (supplementary figure 5); therefore, we decided to investigate the role of PFKFB3 in DNA repair biological process.

Among the DEGs involved in DNA repair biological process, ATM was changed significantly. DNA damage usually results in G2/M phase arrest by activating cell cycle checkpoint genes, and ATM plays an important role in the cell cycle checkpoint and prevents damaged cells from proceeding to mitosis^17^. We found that ATM and its associated genes, including p21, Chk1, cdc25C, cdc2, and Cyclin B1, were listed in the DEGs. Furthermore, we tested these proteins using Western blot assay and found that PFKFB3 silencing increased the expression of ATM, p21, and Chk1 and inhibited the expression of cdc25C, p-cdc25C, cdc2, and Cyclin B1, and that overexpressed PFKFB3 increased the expression of cdc25C, p-cdc25C, cdc2, and Cyclin B1 and decreased the expression of ATM, p21, and Chk1 (Fig. 4b), suggesting that knockdown PFKFB3 caused G2/M phase arrest that was triggered by cell cycle checkpoint genes, followed by DNA damage.

Accumulating evidence has shown that AKT is a direct participant in the DNA damage response and repair^18^. We found the AKT expression and phosphorylation decreased in PFKFB3 silencing cells (Fig. 4d). An analysis based on the STRING database showed that PFKFB3 and AKT may have a direct interaction^19^. We conducted an immunoprecipitation assay and found that AKT protein was immunoprecipitated by the anti-PFKFB3 antibody (Fig. 4c), suggesting that PFKFB3 physically associates with AKT. To determine the genes regulated by AKT and responsible for DNA repair mechanism, we tested three candidate genes, ERCC1, NRF2, and FoxO3a, which were identified in the aforementioned DEGs and involved in DNA repair by the GO analysis. Western blot assay showed that only ERCC1 decreased in the PFKFB3 silenced cells (Fig. 4d). ERCC1 is an important gene regulating DNA repair^20^, which is a downstream gene of the AKT pathway^18^. Then, we found that PFKFB3 silencing reduced expression of AKT, pAKT, and ERCC1 (Fig. 4d). Finally, we found that inhibition of AKT phosphorylation by MK-2206 in Huh7-PFKFB3 cells did not change the expression of PFKFB3 but did decrease the expression of ERCC1. Furthermore, ATM/Chk1/cdc25C cells were upregulated compared with untreated Huh-PFKFB3 cells (Fig. 4e). And the MK-2206 is an inhibitor of AKT phosphorylation, suggesting that it is an allosteric inhibitor of AKT, and leads to the changes in AKT phosphorylation could be due to the feedback loop.

### PFKFB3 inhibitor PFK15 inhibited HCC growth in vivo and in vitro

**Fig. 5:**
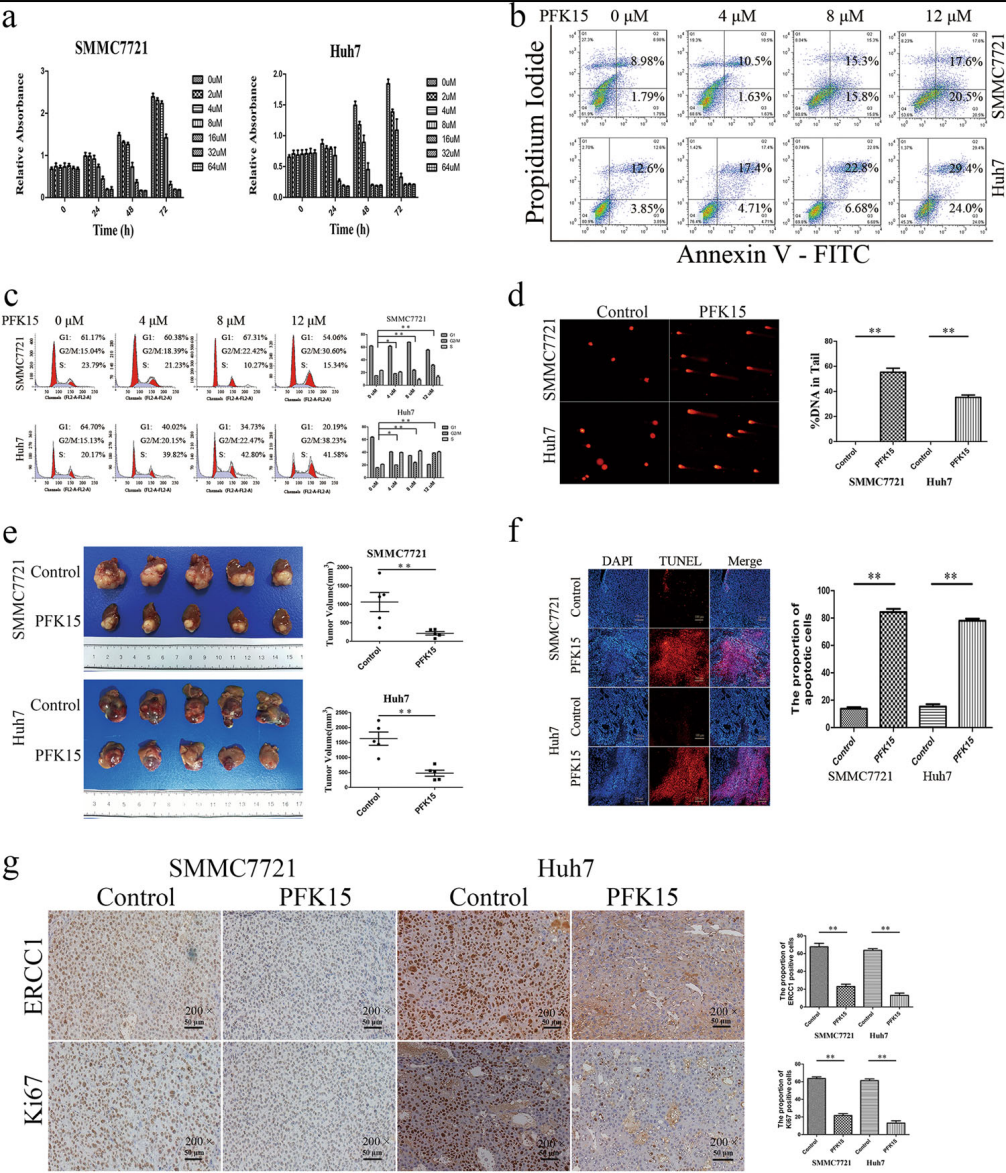
PFKFB3 inhibitor PFK15 inhibits HCC growth in vivo and in vitro. **a** CCK8 assay for cell proliferation of SMMC7721 cells and Huh7 cells treated with PFK15 in a dose and time series. PFK15 inhibited proliferation of SMMC7721 and Huh7 in a dose-dependent and time-dependent manner. **b** Flow cell apoptosis detection of the cell apoptosis rate of SMMC7721 cells and Huh7 cells treated with PFK15 in a dose and time series. PFK15 increased the apoptosis rate of SMMC7721 and Huh7 in a dose-dependent and time-dependent manner. **c** Flow cytometry cycle detection of the cell cycle ratio of SMMC7721 cells and Huh7 cells treated with PFK15 in a dose and time series. PFK15 induced G2/M arrest of SMMC7721 and Huh7 in a dose-dependent and time-dependent manner. **d** Comet assay for DNA damage of SMMC7721 cells and Huh7 cells treated with PFK15 (24 h). PFK15 led to DNA damage in vitro. **e** Comparison of tumor sizes of the SMMC7721-control group and SMMC7721-PFK15 group (1062.2 ± 578.2 mm^3^ vs. 215.9 ± 104.8 mm^3^; *p* = 0.03), and comparison of tumor sizes of the Huh7-control group and Huh7-PFK15 group (1628.4 ± 495.5 mm^3^ vs. 475.4 ± 222.9 mm^3^; *p* = 0.004). PFK15 treatment delayed tumor growth in both SMMC7721 and Huh7 tumor models. **f** Representative immunohistochemistry of liver cancer showing the expressions of Ki67 and ERCC1 from Balb/c nu/nu mice orthotopically implanted with SMMC7721 or Huh7 cells. PFK15 treatment decreased the expression of ERCC1 and Ki67.(Magnification ×200). **g** Representative TUNEL fluorescence of liver cancer from Balb/c nu/nu mice orthotopically implanted with SMMC7721 or Huh7 cells. PFK15 treatment increased the apoptosis rate of tumor.(Magnification ×200). **p < 0.01

1-(4-pyridinyl)-3-(2-quinolinyl)-2-propen-1-one (PFK15) is a small molecule competitive PFKFB3 inhibitor for which a phase I clinical trial of advanced solid tumors was initiated in 2013^21^. In this study, we found that PFK15 treatment inhibited proliferation of SMMC7721 and Huh7 in a dose-dependent and time-dependent manner (Fig. 5a), increased the apoptosis rate of SMMC7721 and Huh7 (Fig. 5b), induced G2/M arrest (Fig. 5c), and led to increased DNA damage (Fig. 5d) in vitro.

In an animal study, PFK15 treatment significantly delayed tumor growth in both SMMC7721 and Huh7 tumor models compared with the vehicle-treated group (Fig. 5e). An immunohistochemistry assay demonstrated that apoptosis was increased in tumor cells(Fig. 5f) and ERCC1 expression and cell proliferation (Ki67 as a marker) were decreased (Fig. 5g) in the PFK15-treated tumors compared with the vehicle-treated tumors.

## Discussion

The present study demonstrated that PFKFB3 expression, which is mainly located in the nucleus of HCC cells, was associated with patient survival. Furthermore, PFKFB3 not only promoted glucose consumption in HCC cells but also functioned as an important regulator of cell cycles. Inhibition of PFKFB3 induced downregulation of ERCC1 followed by accumulated DNA damage, resulting in G2/M arrest and tumor growth delay.

In the study of PFKFB3 expression and clinical features of patients, we found that PFKFB3 expression was associated with tumor size and patient survival and played an important role in tumor growth. The results were in accordance with studies of other cancers^21^. However, the study of PFKFB3 has mainly focused on its role in the metabolic pathways of cancer cells^22,23^. In the present study, we found that PFKFB3 was mainly located in the nucleus and less so in cytoplasm in HCC cells; several other studies reported similar findings for other types of cancer cells^12^. Therefore, we intended to explore the clinical implications and mechanisms of PFKFB3 expression in HCC.

Two mechanisms may involve the suppression of tumor cell proliferation by inhibition of PFKFB3 expression. One is downregulation of glycolysis and reduction of energy supply to the cells. The other is direct upregulation of cell proliferation, which mainly occurs in the nucleus. To eliminate the effects of PFKFB3 on glycolysis, we used pyruvate sodium containing glucose-free medium as the glucose substitute medium and proved that tumor cell proliferation was not different in the two types of culture medium.

We found that PFKFB3 silencing inhibited proliferation, increased the apoptosis rate, and induced the G2/M phase arrest of HCC cells when the cells were cultured in the glucose substitute medium. Results from the present study showed that DNA damage was evident in the PFKFB3 silenced cells and that several cell cycle checkpoint genes were induced, consistent with the report showing that DNA damage was the primary cause of G2/M phase arrest^16^. To explore why DNA damage is evident in the PFKFB3 silenced cells, we performed a cDNA array assay and found that DNA repair-associated biological processes were one of the most affected biological processes, and the PI3 kinase–Akt pathway was enriched by DEGs, which is in line with findings that showed that AKT is a direct participant in DNA damage response and repair^18^. We also found the AKT expression and phosphorylation both decreased in the PFKFB3 silenced cells and proved that PFKFB3 protein had a direct interaction with AKT protein, suggesting that PFKFB3 directly regulated AKT expression and phosphorylation. Then, we found that ERCC1 may be the responsible gene for DNA repair and was regulated by PFKFB3 expression, and that ERCC1 is one of the important genes involved in DNA repair^24,25^. A study showed that PFKFB3 silencing in Hela cells may activate TP53-induced glycolysis and the apoptosis regulator (TIGAR)-mediated pro-survival pathway, but the net effect of PFKFB3 silencing is to diminish cell proliferation^26^. On the contrary, transforming growth factor-β may upregulate PFKFB3 expression via the PI3K–Akt pathway^27^, suggesting that reciprocal regulation may exist between the PI3K–Akt pathway and PFKFB3.

Various exogenous and endogenous factors act on DNA and lead to DNA injuries^28,29^. With cancer, chemotherapeutics may produce therapeutic effects through damage to DNA that then results in cell death^30^. However, it has been proven that DNA repair in tumor cells is the main cause for chemotherapy failure^31^; therefore, targeting PFKFB3 may reduce glucose metabolism and overcome resistance to chemotherapy, suggesting a combination treatment using PFKFB3 and chemotherapeutics for cancer.

Our study also had some limitations. Many biological processes were associated with PFKFB3 silencing in HCC cells, but only DNA repair was explored based on the findings (G2/M phase arrest). The roles of PFKFB3 in protein synthesis^32^ and cell reprogramming^27^ were not focused on in the present study. We found that some other proliferation signaling pathways, like the Hippo pathway, were also associated with PFKFB3 silencing, but we did not explore them.

In conclusion, this study showed that PFKFB3 expression promotes HCC growth through the PFKFB3/AKT/ERCC1 signaling pathway to enhance the ability of DNA repair and its pro-tumor effects during glycolysis. The expression of PFKFB3 was correlated with a poor prognosis for HCC patients. PFKFB3 inhibition inhibited tumor growth, suggesting that PFKFB3 may be a potential target for HCC treatment and may play a role in overcoming resistance to chemotherapy.

## Materials and methods

### Cell cultures

The SMMC7721, Huh7, MHCC-LM3, MHCC-97H, MHCC-97L, Hep 3B 2.1-7, and Hep G2 cell lines are human HCC cell lines. L02 is a hepatocyte cell line. All cell lines were obtained from the Liver Cancer Institute at Fudan University in Shanghai, China. Cells were cultured in high-glucose DMEM supplemented with 10% fetal bovine serum (FBS; Gibco, Gaithersburg, MD, USA) and 1% penicillin/streptomycin or glucose-free DMEM plus sodium pyruvate (0.1 mol/L) with 10% FBS (Gibco) and 1% penicillin/streptomycin under 5% CO_2_ at 37 degree centigrade.

### Knockdown and overexpression of PFKFB3 by transfection

GPH-h-PFKFB3-sh, GTP-H-PFKFB3 lentivirus, and vector control were constructed by Bio-link-gene Co., Ltd. (Shanghai, China); 3 × 10^5^ cells were seeded in each well of a six-well plate. Then, the lentiviruses were added to the well with 1 ml of DMEM containing no FBS and 5 μg/ml Polybrene (Sigma, USA). Twelve hours later, medium containing the virus was removed and replaced with medium containing 10% FBS. Then, expression of PFKFB3 was validated by qPCR and Western blot.

### Immunohistochemistry

Tissue microarrays (TMAs) contained tumor and non-tumor specimens from 287 HCC patients after curative resection between 2005 and 2006 at our institute. The use of tumor tissues was approved by the Ethics Committee of Zhongshan Hospital at Fudan University, and informed consent was obtained from each patient. Patient follow-up was conducted as previously described^33^. The time interval between resection and death or last follow-up date was defined as OS, and the time interval between resection and tumor recurrence or death was defined as DFS.

The TMA was created as described previously^34^ and was incubated with rabbit antibody anti-PFKFB3 (1:100, Abcam, UK). An Ultra-Vision Quanto Detection System HRP DAB (Thermo Fisher Scientific, CA, USA) was used to detect PFKFB3 expression. Integrated optical density (IOD) was assessed using Image-Pro Plus 6.0 software^35^. According to the IOD, patients were divided into low (*n* = 143) or high (*n* = 144) PFKFB3 expression groups.

### Western blot and immunoprecipitation assay

Total protein was extracted with RIPA lysis buffer with protease inhibitor from HCC cell lines or tissues; 30 μg of total protein subjected to Western blot were separated using 10% SDS-PAGE and electro-transferred onto polyvinylidene difluoride membranes (Millipore, Billerica, MA, USA). Membranes were blocked with 5% skim milk and then incubated with the primary antibody.

Mouse antibody for β-actin was purchased from Beyotime (Jiangsu, China). The rabbit antibodies for PFKFB3 were purchased from Abcam (Cambridge, UK). The rabbit antibodies for Cyclin B1, P21, AKT, and pAKT were purchased from Cell Signaling Technology (USA). The rabbit antibodies for CDC2, cdc25C, Chk1, and ERCC1 were purchased from Proteintech (Chicago, IL, USA). The rabbit antibodies for pcdc25C were purchased from Sab-biotech (USA). The rabbit antibody for ATM and pATM was purchased from Zen-bio (Chengdu, China).

For immunoprecipitation, cell lysis was performed using lysis buffer and the supernatant was collected after centrifugation. Antibody was added to lysates with protein A beads and incubated overnight. The beads were collected and subjected to Western blot.

### Quantitative real-time PCR

Quantitative real-time PCR was performed on RNA extracted from HCC cell lines or tissues. Total RNA was isolated according to the manufacturer’s protocol. The concentration and purity of all RNA samples were determined using the A260–A280 nm ratio. The following primers were used: PFKFB3, 5′-GGT CGG AAG AGT GGA CTT TG-3′ (forward) and 5′-CAG GGT TTG AGG CAA TGA G-3′ (reverse); and β-actin, 5′-AAG GTG ACA GCA GTC GGT T-3′ (forward) and 5′-TGT GTG GAC TTG GGA GAG G-3′ (reverse).

### Microarray analysis

Total RNA was extracted from SMMC7721-shVector and SMMC7721-shPFKFB3 cell lines. The 60-mer oligo nucleotide probes were designed using a microarray (Agilent) and performed by Oe-biotech (Shanghai, China). We determined the differently expressed genes and performed GO and KEGG analysis.

### CCK8 assay

Three thousand cells in 100 μl medium were seeded in a well of a 96-well plate, and measurements were performed 24, 48, and 72 h after seeding according to the manufacturer’s protocol. Cells were incubated in 100 μl reaction mixture (10 μl CCK-8 and 90 μl DMEM) for 2 h and measured at a wavelength of 450 nm.

### Clone formation assay

One thousand cells were seeded in a well of a six-well plate and cultured under 5% CO_2_ at 37 degree centigrade for 2 weeks. Then, the cells were fixed with formalin for 30 min and stained with 0.1% crystal violet for 15 min.

### Flow cytometry

Cells were washed with phosphate-buffered saline (PBS) and stained with annexin V and propidium iodide or 7-aminoactinomycin (7-AAD) (BD Pharmingen, San Diego, CA, USA). Fluorescence was measured using a FACSCalibur (BD Biosciences, San Jose, CA, USA) and analyzed using FlowJo (Tree Star, Ashland, OR, USA). Annexin V/PI or annexin V/7-AAD cells were quantified by the frequency of fluorescently labeled cells. Statistical significance was assessed by the two-sample *t*-test (independent variable).

### Xenograft model of HCC in nude mice

As described in our previous study^36^, male BALB/c nu/nu mice (5 weeks old) were purchased from the Shanghai Institute of Materia Medical, Chinese Academy of Science, and housed under specific pathogen-free conditions. The experimental protocol was approved by the Shanghai Medical Experimental Animal Care Commission. Mice were randomly assigned to the experimental group and the control group, and various cancer cells (6 × 10^6^ cells) in 200 μl of normal saline were implanted by subcutaneous injection to obtain subcutaneous tumors. Four weeks later, the mice were killed to obtain the tumors, which were then inoculated into the livers of nude mice. Five mice in each group were fed for 4 weeks and then killed.

### TUNEL assay

Apoptosis of orthotopic liver tumors was detected by the In Situ Cell Detection Kit (Roche, Basel, Switzerland) according to the manufacturer’s instruction. Sections were incubated in citrate buffer and heated for 10 min after deparaffinization and rehydration. Then, they were washed twice with PBS. Sections were immersed with 50 μl TUNEL working solution per sample and incubated at 37 degree centigrade for 60 min in the dark. Then, they were washed twice with PBS and analyzed by fluorescence microscopy.

### Comet assay

The alkaline comet assay was performed to detect the level of DNA damage in HCC cell lines (Trevigen, Gaithersburg, MD, USA). Slides were placed on the electrophoresis slide tray. Electrophoresis was conducted using an electric field of 21 V for 30 min in alkaline electrophoresis solution (0.03 M NaOH, pH 13). Then, the slides were immersed twice in distilled water for 5 min each and in 70% ethanol for 5 min. Samples were dried at 37 degree centigrade for 15 min. Finally, the slides were stained with propidium iodide (1 μg/mL) and captured by fluorescence microscopy.

### Statistical analysis

Statistical analysis was performed with SPSS 19.0 for Windows (SPSS Inc., Chicago, IL, USA). Continuous variables were expressed as the mean ( ± standard deviation) or median (interquartile range), as appropriate. Categorical variables were compared using the *χ*^2^ test or Fisher exact test, and continuous variables were compared using the Student *t*-test or Mann–Whitney *U*-test. Univariate survival analysis was performed using the Kaplan–Meier method, and the significance of the difference between the groups was analyzed using the log-rank test. The relative prognostic significance of the variables for predicting OS and RFS was assessed using Cox proportional hazards regression models. All statistical tests were two-tailed, and *P* < 0.05 indicated a significant difference.

## Electronic supplementary material

Supplementary Figure 1(PDF 1108 kb)

Supplementary Figure 2(PDF 1043 kb)

Supplementary Figure 3(PDF 847 kb)

Supplementary Figure 4(PDF 700 kb)

Supplementary Figure 5(PDF 627 kb)

Supplementary Figure Legends(DOCX 55 kb)
