# Supplementary figures and images for: PFKFB3 blockade inhibits hepatocellular carcinoma growth by impairing DNA repair through AKT
Source: Cell Death Dis. 2018 Mar 20;9(4):428. doi: 10.1038/s41419-018-0435-y (PMC5861039; doi:10.1038/s41419-018-0435-y)

A

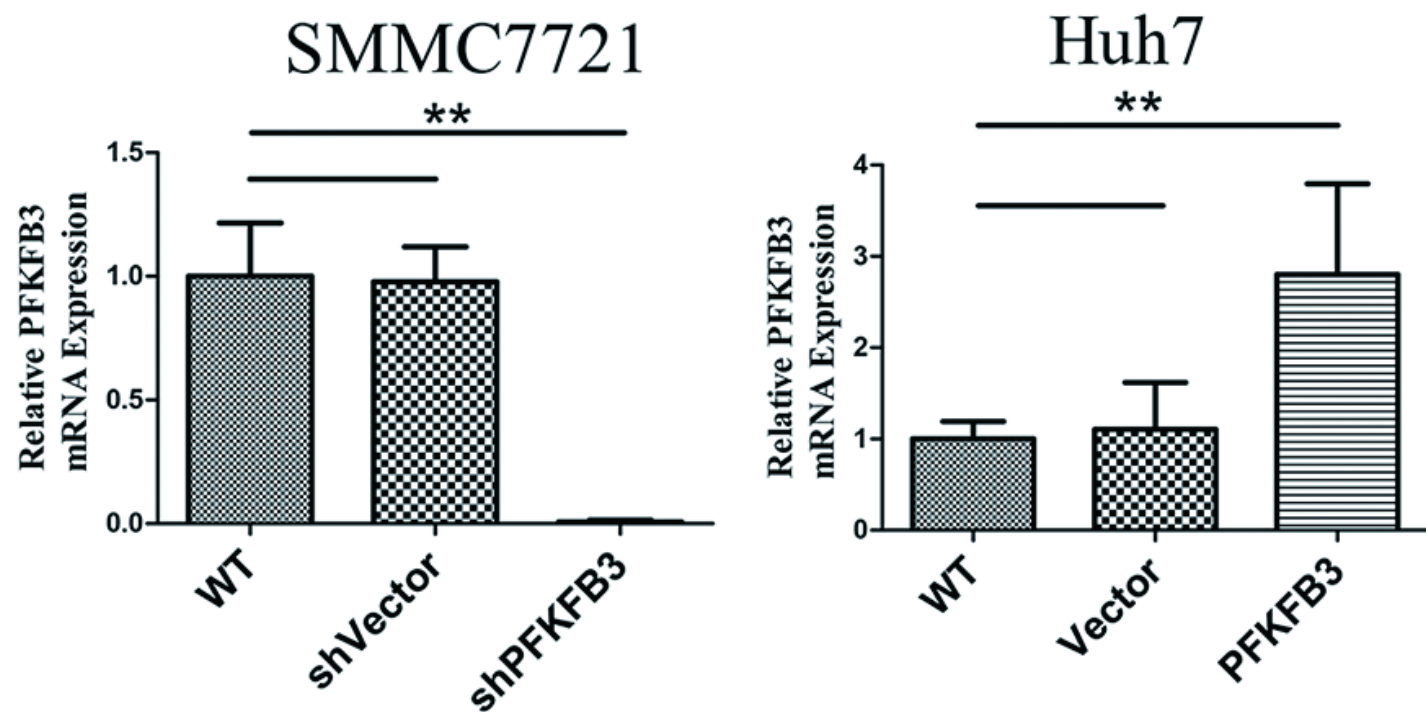

B

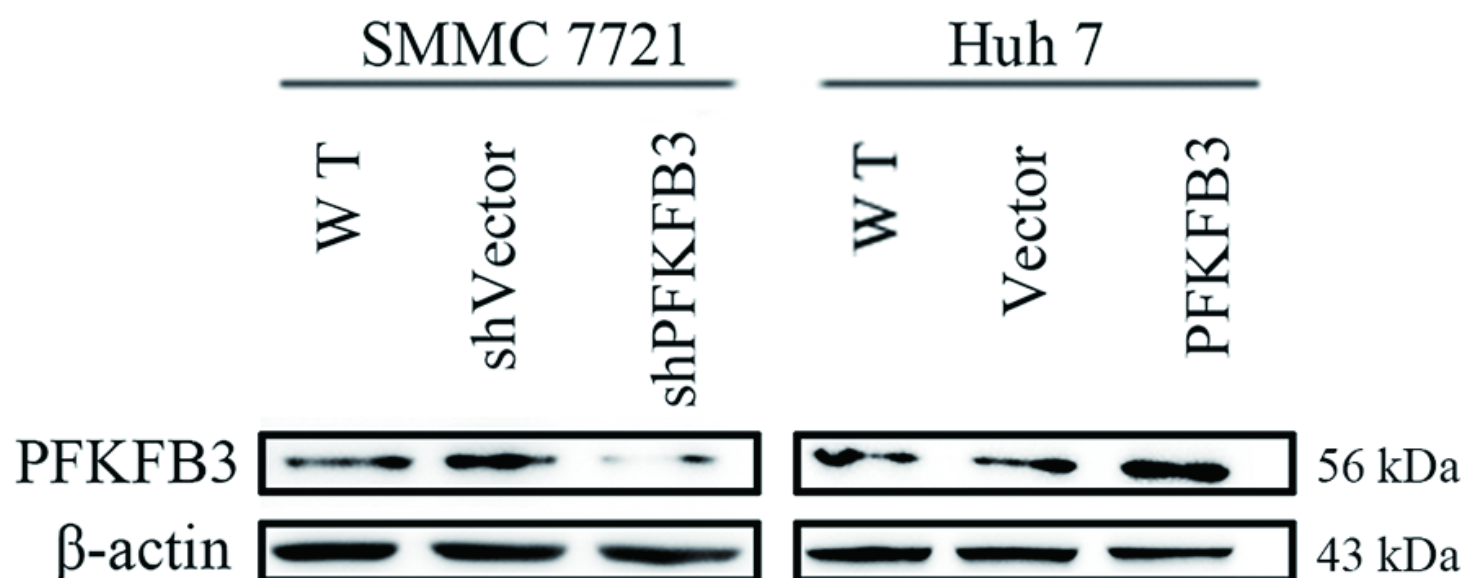

Supplement: Supplementary file 1 — Supplementary Figure 1(PDF 1108 kb) [file 41419_2018_435_MOESM1_ESM.pdf]

SMMC7721

shVector

shPFKFB3

DAPI

PFKFB3

Merge

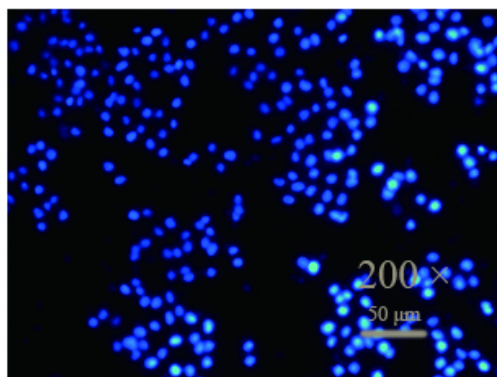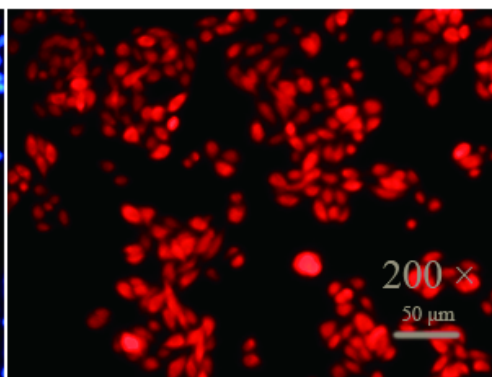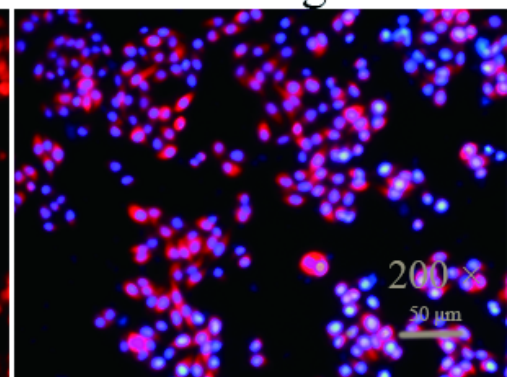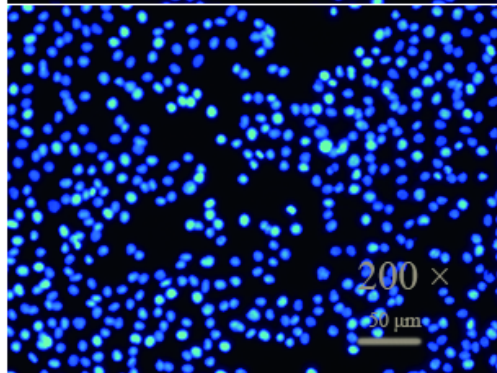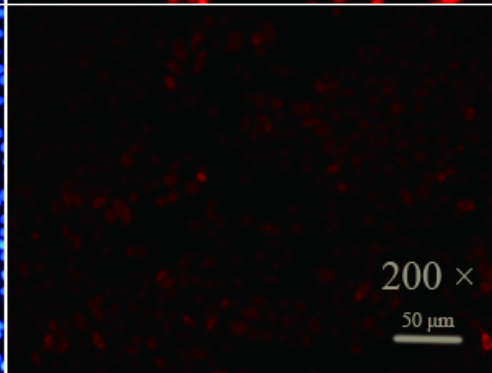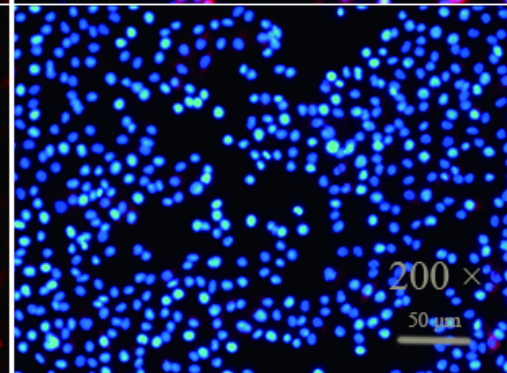

Supplement: Supplementary file 2 — Supplementary Figure 2(PDF 1043 kb) [file 41419_2018_435_MOESM2_ESM.pdf]

A

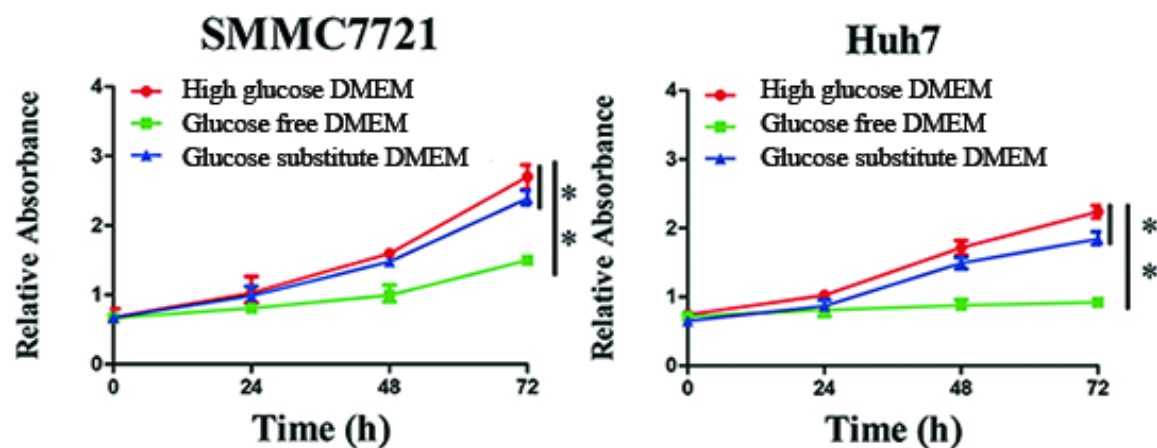

B

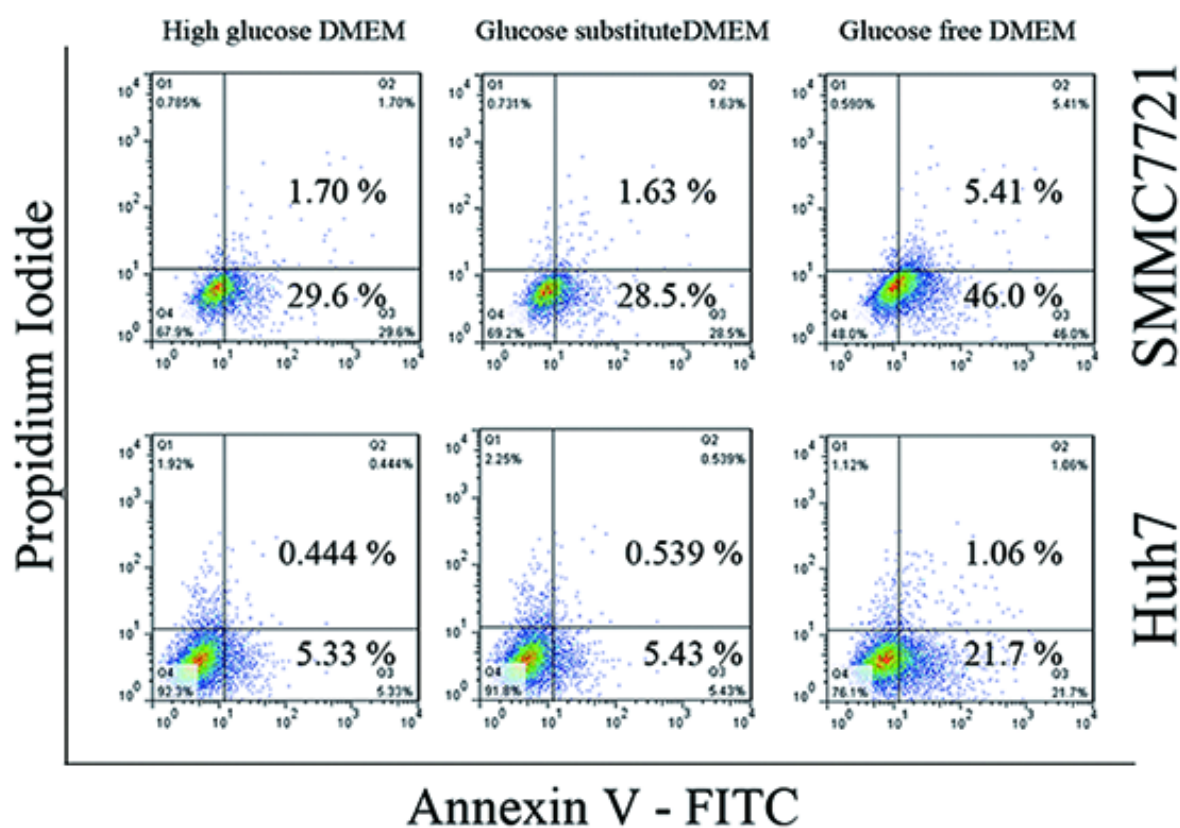

C

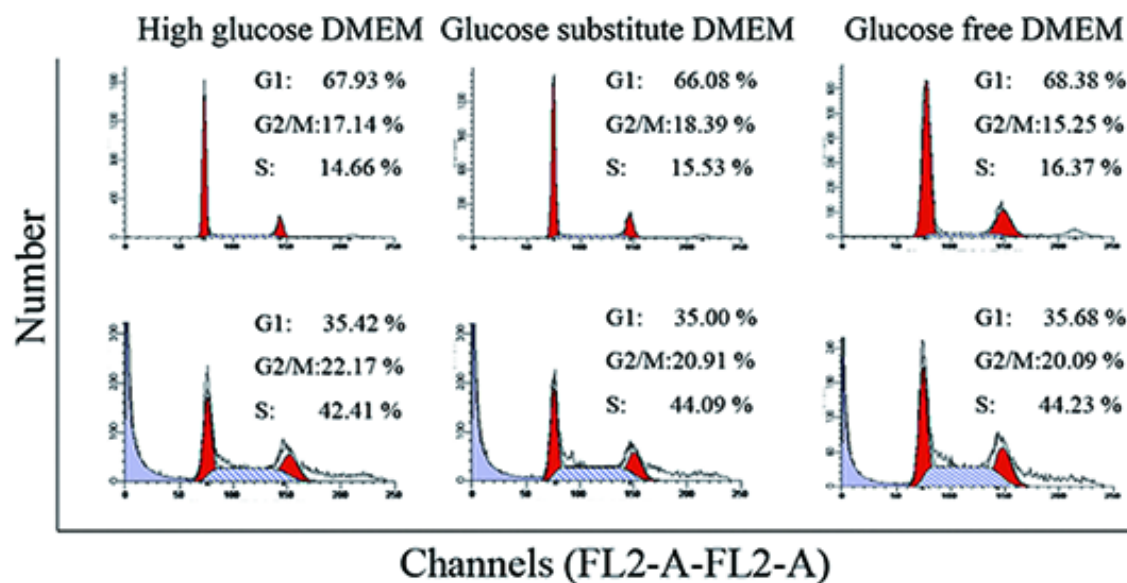

Supplement: Supplementary file 3 — Supplementary Figure 3(PDF 847 kb) [file 41419_2018_435_MOESM3_ESM.pdf]

KEGG

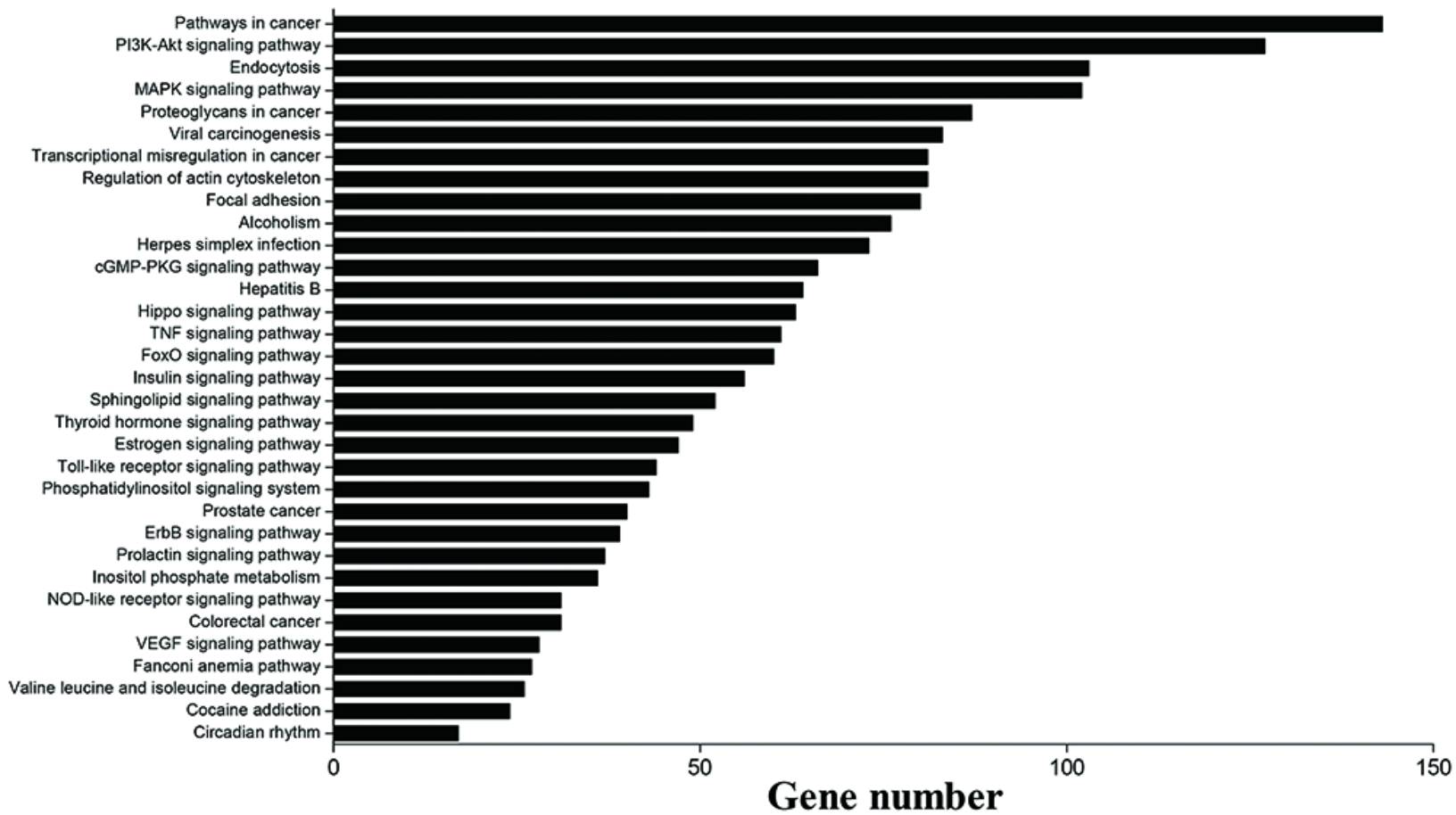

Supplement: Supplementary file 4 — Supplementary Figure 4(PDF 700 kb) [file 41419_2018_435_MOESM4_ESM.pdf]

# GO-Biological Process

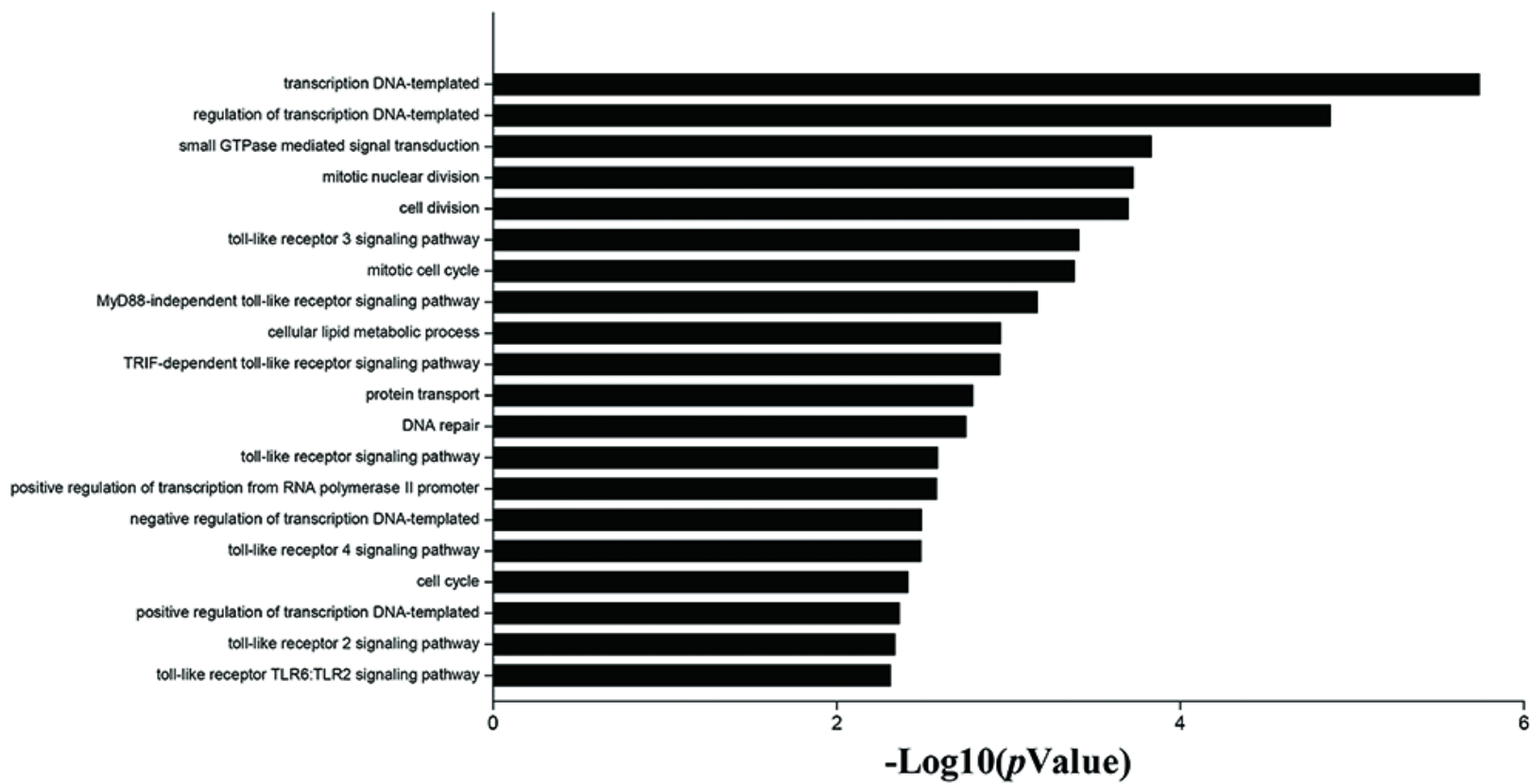

Supplement: Supplementary file 5 — Supplementary Figure 5(PDF 627 kb) [file 41419_2018_435_MOESM5_ESM.pdf]
