# Supplementary material for: PFKFB3 blockade inhibits hepatocellular carcinoma growth by impairing DNA repair through AKT
Source: Cell Death Dis. 2018 Mar 20;9(4):428. doi: 10.1038/s41419-018-0435-y (PMC5861039; doi:10.1038/s41419-018-0435-y)
Supplement: Supplementary file 6 — Supplementary Figure Legends(DOCX 55 kb) [file 41419_2018_435_MOESM6_ESM.docx]

**PFKFB3 blockade inhibits hepatocellular carcinoma growth by impairing DNA repair through AKT**

**Supplementary Figure Legends**

Supplementary figure 1. The detection of different PFKFB3 expression in cells transfected with lentiviral vectors. (A)Western blot analysis of PFKFB3 protein expression in SMMC7721-shVector, shPFKFB3 and Huh7-Vector, PFKFB3 cells. PFKFB3 protein expressed higher in Huh7-PFKFB3 than in Huh7-Vector and expressed lower in SMMC7721-shPFKFB3 than in SMMC7721-shVector. (B) qRT-PCR expression analysis of PFKFB3 mRNA in SMMC7721-shVector, shPFKFB3 and Huh7-Vector, PFKFB3 cells. SMMC7721 PFKFB3 knockdown cell line and Huh7 overexpressed cell line constructed successfully. PFKFB3 mRNA expressed higher in Huh7-PFKFB3 than in Huh7-Vector and expressed lower in SMMC7721-shPFKFB3 than in SMMC7721-shVector.

Supplementary figure 2. The detection of PFKFB3 protein expression in SMMC7721-shPFKFB3 by IF. PFKFB3 knockdown reduced the PFKFB3 expression in nucleus.

Supplementary figure 3. The comparition of different culture media on proliferation, apoptosis and cell cycle of hepatoma cell lines. (A) CCK8 assay for cell proliferation of SMMC7721 and Huh7 cells in high-glucose DMEM, glucose-free DMEM, and glucose substitute DMEM. Cell proliferation decreased in glucose free medium, but changed unconspicuously in glucose-substitute DMEM, compared with in high glucose DMEM. (B) Flow cell apoptosis detection for SMMC7721 and Huh7 cells in high-glucose DMEM, glucose-free DMEM, and glucose substitute DMEM. Cell apoptosisrate increased in glucose free medium, but changed unconspicuously in glucose-substitute DMEM, compared with in high glucose DMEM. (C) Flow cytometry cycle detection for SMMC7721 and Huh7 cells in high-glucose DMEM, glucose-free DMEM, and glucose substitute DMEM. Cell cycle changed unconspicuously in three different culture medium.

Supplementary figure 4. The changed signaling pathway of SMMC7721-shPFKFB3 cells. The signaling pathways were selected based on the *P* value (<0.05) and then ranked by the number of different expression genes that enriched the pathway.

Supplementary figure 5. The changed biological processes in SMMC7721-shPFKFB3 cells (top 20). There were 100 changed biological processes (*P* < 0.05). We selected the first 20 biological processes that were ranked by the -Log_10_ (*P* value).
